# Pesticides and Neurologic Symptoms

**DOI:** 10.1289/ehp.113-1314952

**Published:** 2005-12

**Authors:** Carol Burns, Daniel A. Goldstein

**Affiliations:** The Dow Chemical Company, Midland, Michigan, E-mail: cburns@dow.com; The Monsanto Company

We read with interest the recent study titled “Neurologic Symptoms in Licensed Private Pesticide Applicators in the Agricultural Health Study” (Kamel et al. 2005). Although this was a hypothesis-generating study, the authors speculated regarding moderate exposure and associations with neurologic symptoms. Substantiation of hypotheses requires meaningful metrics of exposure and effect, and depends on exclusion and analysis of competing hypotheses for the observations. In our opinion, the article by Kamel et al. falls seriously short in several regards and requires additional data in order to provide credible and defensible conclusions.

Kamel et al. (2005) analyzed a number of symptoms in those “ever” experiencing one of 23 self-reported symptoms in the preceding 12 months. The biologic significance of the outcome “symptom count” is unknown; also, “multiple symptoms” is not a definable disease or illness. The fact that private applicators report headache, nausea, and fatigue does not establish that each is of neurologic origin, particularly given the physical requirements of farming. Indeed, results of the questions used by Kamel et al. (2005) have been shown to agree poorly with objective tests of neurologic function (Lundberg et al. 1997). Further, Kamel et al. limited the analyses to a single episode rather than symptoms that were reported more than once per year (Kamel et al. (2005); Table 2). As a cross-sectional analysis, the data do not permit assessment of the temporal relationship between exposure and symptom onset, and no consideration was given to the transient nature of the reported symptoms. Thus, although the nature of the analysis implies some sort of persistent neurologic condition underlying the reporting of symptoms, no such condition can be established from intermittent symptoms of indeterminate etiology.

In addition to other potential causes for these symptoms, researchers have warned about the role of psychosocial factors in the reporting of non-specific symptoms. According to Spurgeon et al. (1996),

Many occupational and environmental health hazards present as an increased reporting of non-specific symptoms such as headache, backache, eye and respiratory irritation, tiredness, memory problems, and poor concentration. The pattern and number of such symptoms is surprisingly constant from hazard to hazard suggesting that common psychological and social factors, not directly related to the exposure may be involved.

The role of these factors has been well documented in the psychological literature. Such factors include attitudes and belief systems; current or preexisting stress; workers’ perception of the competence and credibility of management; and involvement of the media, pressure groups, and the legal system (Spurgeon et al. 1997). Further, “[p]revention and control strategies are unlikely to be successful if the real sources of the problems are not correctly identified” (Spurgeon et al. 1997).

Because Kamel et al. (2005) relied on self-reported days of application to infer exposure rather than actual measured dose, their assumption of sufficient exposure to cause a biologic effect has severe limitations. The Agricultural Health Study (AHS) has used lifetime exposure days for specific, individual pesticides in other publications (Alavanja et al. 2003, 2004; Engel et al. 2005), but Kamel et al. (2005) offered no support for their change in approach and the validity of a class-wide, rather than pesticide-specific biologic effect. Furthermore, studies indicate that farmers have much less pesticide exposure than is often assumed from self-reported use and even within this low range; the exposure is variable for a given day. For example, in a study of organophosphate applicators, Stokes et al. (1995) identified differences in urinary metabolite levels based on the number of tanks loaded, acres sprayed, and hours sprayed. Other bio-monitoring studies have identified a large range of exposure for different pesticides, including applicators with no detectable exposure (Arbuckle et al. 2002; Mandel et al. 2005). The exposure metric used by Kamel et al. (2005) of cumulative lifetime days applied most likely overestimates exposur, in light of these exposure studies of farmer applicators.

We believe that the findings of Kamel et al. (2005) may well be the result of evaluating multiple pesticides as groups at a time in conjunction with other physical or emotional stress related to farming or even a common ailment such as influenza (Dunn et al. 1995). In any event, the conclusions are not justified by the data because there is no coherent disease outcome and no meaningful exposure metric. It is our view that even hypotheses generated by such non-specific data do not meet the stated AHS objective, which is to “provide information that agricultural workers can use in making decisions about their health and the health of their families” (AHS 2005).
